# Involvement of brain-derived neurotrophic factor (BDNF) in chronic intermittent stress-induced enhanced mechanical allodynia in a rat model of burn pain

**DOI:** 10.1186/s12868-019-0500-1

**Published:** 2019-04-24

**Authors:** Natasha M. Sosanya, Thomas H. Garza, Winfred Stacey, Stephen L. Crimmins, Robert J. Christy, Bopaiah P. Cheppudira

**Affiliations:** Battlefield Pain Management Research Group, United States Army Institute of Surgical Research, 3698 Chambers Pass, JBSA Fort Sam Houston, San Antonio, TX 78234-4504 USA

**Keywords:** Chronic intermittent stress, Thermal injury, Mechanical allodynia, BDNF, TrkB, p-TrkB, Cyclotraxin-B

## Abstract

**Background:**

Reports show that stressful events before injury exacerbates post-injury pain. The mechanism underlying stress-induced heightened thermal pain is unclear. Here, we examined the effects of chronic intermittent stress (CIS) on nociceptive behaviors and brain-derived nerve growth factor (BDNF) system in the prefrontal cortex (PFC) and hypothalamus of rats with and without thermal injury.

**Results:**

Unstressed rats showed transient mechanical allodynia during stress exposure. Stressed rats with thermal injury displayed persistent exacerbated mechanical allodynia (*P* < 0.001). Increased expression of BDNF mRNA in the PFC (*P* < 0.05), and elevated TrkB and p-TrkB (*P* < 0.05) protein levels in the hypothalamus were observed in stressed rats with thermal injury but not in stressed or thermally injured rats alone. Furthermore, administration of CTX-B significantly reduced stress-induced exacerbated mechanical allodynia in thermally injured rats (*P* < 0.001).

**Conclusion:**

These results indicate that BDNF-TrkB signaling in PFC and hypothalamus contributes to CIS-induced exacerbated mechanical allodynia in thermal injury state.

**Electronic supplementary material:**

The online version of this article (10.1186/s12868-019-0500-1) contains supplementary material, which is available to authorized users.

## Background

Burn injury-induced pain is complex. It originates from the wounds created by a thermal insult and typically leads to excruciating pain during treatment procedures such as debridement, wound dressing, grafting, and wound closure [1, 2]. Burn survivors often experience lingering pain long after discharge from the hospital. Indeed, one study reports a prevalence rate for burn pain as high as 52% for an average of 12 years, demonstrating that burn pain can persist in a chronic state long after wound healing [3]. The underlying neurobiological mechanisms and psychosocial factors that impact post-burn pain are not fully understood. Preclinical studies have shown that exposure to stressors before [4] or after [5] induction of injury alters nociceptive transmission resulting in significant changes in pain behaviors. In addition, the stressors alter the brain and spinal cord neurocircuits and neurochemistry which are associated with stress-mediated nociception [4].

It is known that BDNF plays a critical role in the stress response as evidenced by its altered expression in the brain of stressed animals [6, 7]. BDNF has also been implicated in neuropathic and inflammatory pain mechanisms [8, 9]. BDNF mediates its biological functions [10] through two transmembrane receptors: p75NTR (pan-selective p75 neurotrophin receptor) and the TrkB receptor (tropomyosin receptor kinase B or tyrosine receptor kinase B). BDNF binds with high affinity to TrkB modulating the stress response [11, 12] and nociceptive neurotransmission [8, 13, 14]. Spinal BDNF-TrkB signaling has been implicated in increased pain signaling mechanisms which can be reversed by intrathecal administration of a TrkB antagonist [15]. Reports have shown that the functionality of the hypothalamus and prefrontal cortex (PFC) regions of the brain are essential for stress and pain responses [16–19]. BDNF is highly expressed in these regions and its expression significantly changes in response to chronic pain [20] and stress [6]. However, it is not clear how BDNF signaling is involved in a combined model of chronic stress and burn pain.

Forced swim, sound, restraint, and cold are potential stressors that can significantly induce stress [21–25]. Previous reports show that some of these stressors can also influence nociceptive transmission in experimental animals [21–25]. For example, a recent study from our laboratory and also earlier reports demonstrate that exposure to sound stress prior to injury can exacerbate post-injury pain behaviors in animals [23]. Similarly, the forced swim stressor alters post-stress nociceptive behaviors [22]. However, the cumulative effect of these multiple stressors combined with thermal injury on post-burn pain has not been reported. In the present study, we have developed a chronic intermittent stress (CIS) protocol by utilizing the stressors listed above. We studied the effect of CIS on nociceptive behaviors in thermally injured and uninjured rats. Additionally, we examined the involvement of BDNF-TrkB receptor signaling in the hypothalamus and in the PFC of these rats.

## Results

### Time-dependent changes in basal nociceptive behaviors during and after exposure to CIS procedure in uninjured rats

**Fig. 1:**
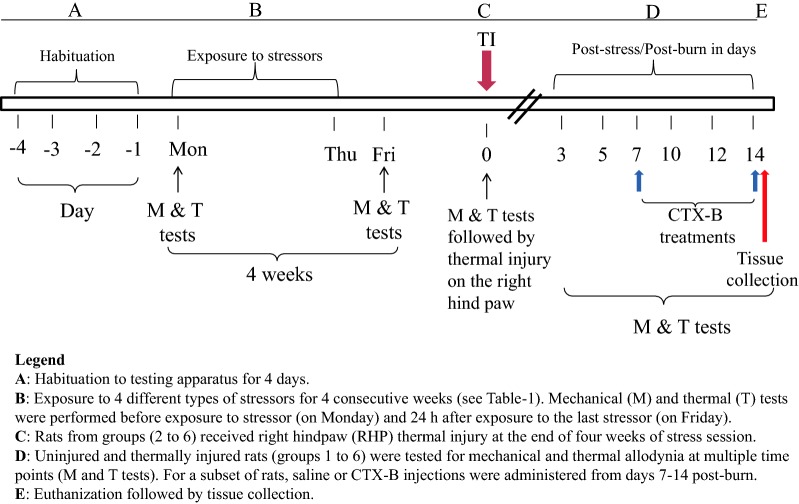
Schematic representation of the experimental design and timeline

**Fig. 2:**
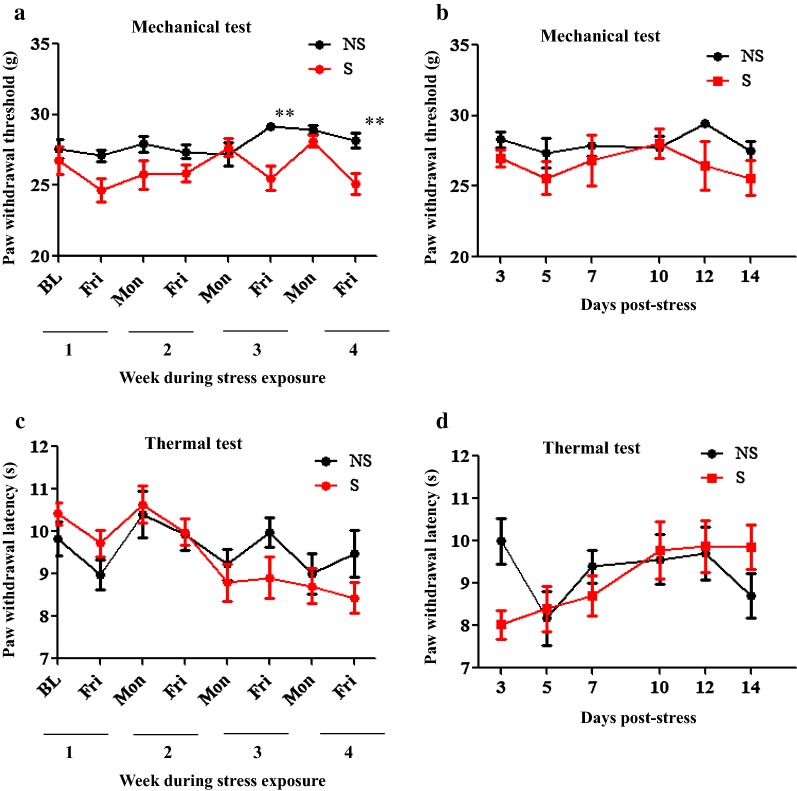
Effects of CIS on mechanical and thermal nociception in uninjured rats. A transient reduction in sensitivity to mechanical stimulus was observed in stressed rats at week 3 and week 4 during exposure to chronic intermittent stress. ** = *P* < 0.01 indicates significant difference between NS and S groups (**a**). After cessation of stress exposure the mechanical threshold was comparable between NS and S groups (*P* > 0.05) (**b**). Thermal threshold between NS and S was not significantly altered during stress session (**c**) or in post-stress period (*P* > 0.05) (**d**). *NS* non-stress, *S* stress. n = 18/group. Data is represented as mean ± SEM

The effect of CIS on nociceptive threshold in uninjured rats was examined. Rats were exposed to chronic stress for 4 weeks (Fig. 1). We combined the left and right hind paws PWT and PWL values for analysis. Two-way RM ANOVA showed F_(1, 34)_ = 11.4, *P* = 0.0019; F_(7, 238)_ = 3.333, *P* = 0.0021; F_(7, 238)_ = 2.59, *P* = 0.0019 for the condition, time, and for the interaction condition × time, respectively, Fig. 2a. Significant decreases in PWTs in response to mechanical stimulation was first observed in S rats compared to NS rats at week 3 (*Post hoc* test, *P* < 0.01) and was also observed at week 4 (*Post hoc* test, *P* < 0.01) during 4 weeks of stress session. No significant changes in PWT between S and NS groups were observed at any times of behavioral testing during post-stress days (all F’s < 2.9, all *P*’s > 0.05, Fig. 2b). Collectively, these results indicate that CIS produced mechanical allodynia is transient in uninjured rats.

Analysis of PWLs during exposure to CIS procedure showed no significant changes in condition (F_(1, 34)_ = 0.1916, *P* = 0.6644) and interaction condition × time (F_(7, 238)_ = 1.841, *P* = 0.0803), but detected significant changes in time (F_(7, 238)_ = 5.87, *P* < 0.0001), Fig. 2c. On post-stress days, Fig. 2d, significant changes in time (F_(5, 80)_ = 3.219, *P* = 0.0107) and interaction x time (F_(5, 80)_ = 3.128, *P* = 0.0125) was observed but no changes in condition (F_(1, 16)_ = 0.07966, *P* = 0.7814). This data suggest that the CIS regimen used in the present study is ineffective to influence basal thermal nociception in rats.

### CIS exacerbates mechanical allodynia in thermally injured rats

**Fig. 3:**
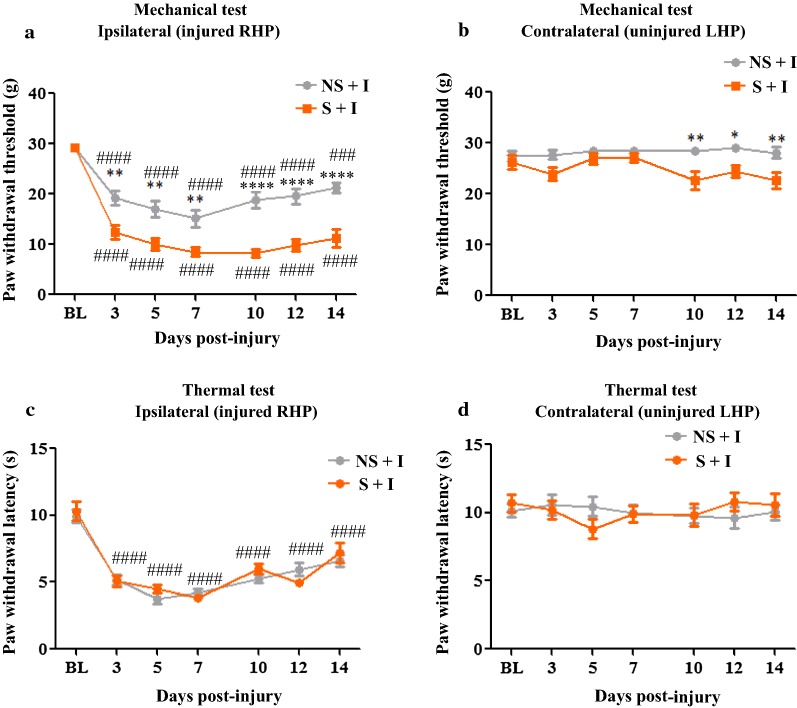
CIS procedure exacerbates mechanical allodynia without effecting thermal allodynia in thermally injured rats. Thermal injury to ipsilateral paw produced time-dependent reduction in withdrawal threshold to mechanical stimulus in NS + I and S + I groups. However, S + I group showed enhanced mechanical allodynia. ^####^ = *P* < 0.0001 indicates significant difference when compared to their respective baseline threshold. ** = *P* < 0.01 and **** = *P* < 0.0001 indicates significant differences between NS + I and S + I groups (**a**). At 10, 12, and 14 days post-thermal injury, the uninjured contralateral paw of rats from S + I group showed significant decrease in PWT compared to the NS + I group. * = *P* < 0.05 and ** = *P* < 0.01 indicates significant difference between NS + I and S + I groups (**b**). Thermal injured ipsilateral paw from NS + I and S + I groups showed significant decrease in PWL compared to their respective baseline values. The changes were observed throughout the testing period. No significant change in PWL between NS + I and S + I groups occurred. ^####^ = *P* < 0.0001 indicates significant difference compared to baseline PWL (**c**). In contralateral paw no significant changes in PWL between NS + I and S + I groups were observed (**d**). *NS* non-stress; *S* stress; *I* Injury. n = 9/group. Data is represented as mean ± SEM

The impact of 4 weeks of CIS procedure on thermal injury-induced mechanical and thermal allodynia was examined at multiple times between days 3–14 post-injury. The baseline mechanical thresholds of ipsilateral paws from S + I and NS + I groups were comparable before exposure to CIS protocol and thermal injury induction: however, after injury, the ipsilateral paws of rats from both NS + I and S + I groups showed a significant decrease in PWT compared to their respective baseline PWTs at all times of assessment indicating presence of persistent mechanical allodynia in both the groups (Fig. 3a). However, rats that were prior exposed to CIS procedure (S + I group) showed a marked reduction in PWT compared to NS + I rats at the corresponding time of behavioral testing demonstrating stress-induced exacerbation of post-burn mechanical allodynia (Two-way RM ANOVA: F_(1, 25)_ = 112.8, *P* < 0.0001; F_(6, 150)_ = 40.3, *P* < 0.0001; F_(6, 150)_ = 3.905, *P* = 0.0012 for the condition, time, and for the interaction condition x time, respectively, Fig. 3a).

CIS exposure also induced mechanical allodynia on an uninjured contralateral paw. The PWT of contralateral paws was not significant between NS + I and S + I groups when measured before CIS exposure and injury (baseline) and until day 7 post-burn, but S + I group exhibited significantly lower PWT than NS + I group between days 10–14 indicating that CIS influences contralateral PWTs at the later stage of injury (F_(1, 25)_ = 18.42, *P* = 0.0002; F_(6, 150)_ = 1.832, *P* = 0.0964; F_(6, 150)_ = 2.012, *P* = 0.0574 for the condition, time, and for the interaction condition x time, respectively, Fig. 3b).

In the thermal test, the ipsilateral paws from NS + I and S + I groups showed a significant reduction in PWL compared to their respective baseline values demonstrating the development of thermal allodynia (Fig. 3c). Furthermore, there was no significant difference in PWLs between NS + I and S + I groups on post-burn days suggesting exposure to CIS protocol before the injury has no effect on post-burn thermal allodynia (Two-way RM ANOVA: F_(1, 25)_ = 0.2178, *P* = 0.6447; F_(6, 150)_ = 47.84, *P* < 0.0001; F_(6, 150)_ = 1.236, *P* = 0.2910 for the condition, time, and for the interaction condition x time, respectively, Fig. 3c). Additionally, no change in contralateral PWLs between stressed and non-stressed animals with thermal injury was observed (Two-way RM ANOVA: All F’s < 1.2 and all P’s > 0.05, Fig. 3d). These data indicate that CIS procedure causes worsening of mechanical allodynia but is ineffective in altering thermal allodynia in burn state.

### Effect of CIS on BDNF, TrkB, and p-TrkB expression in the PFC and hypothalamus

**Fig. 4:**
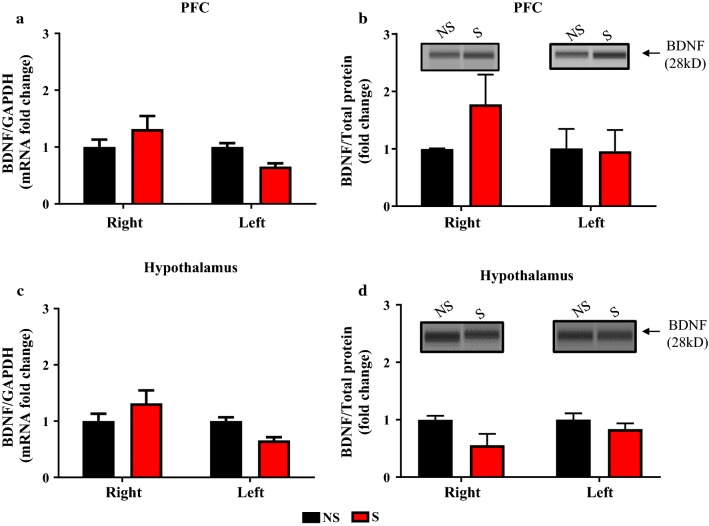
Effect of CIS on PFC and hypothalamus BDNF mRNA and protein levels in uninjured rats. CIS exposure had no significant effect on right and left sides BDNF mRNA and protein expression within and between NS and S groups in the PFC (**a**, **b**) and hypothalamus (**c**, **d**). *NS* non-stress, *S* stress. n = 5/group. Data is presented as mean ± SEM

**Fig. 5:**
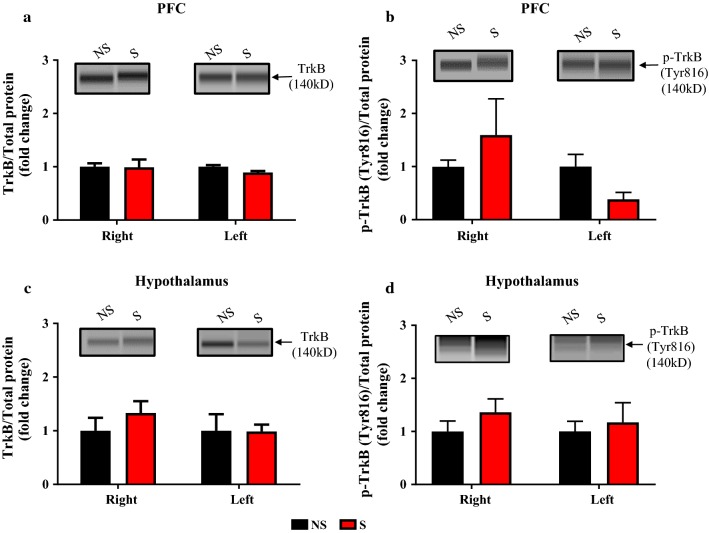
Effect of CIS on PFC and hypothalamic TrkB and p-TrkB protein levels in uninjured rats. CIS exposure had no significant effect on right and left sides TrkB and p-TrkB levels within and between NS and S groups in the PFC (**a**, **b**) and hypothalamus (**c**, **d**). TrkB and p-TrkB protein level in the right and left sides of PFC and hypothalamus are shown by Simple Western blot representative image (above quantification graphs of **a**–**d**). Neither right nor left PFC nor hypothalamic TrkB or p-TrkB levels changed following CIS exposure (**a**–**d**). *NS* non-stress, *S* stress. n = 5/group. Data is presented as mean ± SEM

The influence of CIS on BDNF mRNA and protein expression in PFC and hypothalamus from uninjured rats, day 14 post-stress (Fig. 1 timeline), were examined using RT-PCR and Simple Western methods (Fig. 4). There was no significant difference in BDNF mRNA and protein expressions between right and left sides of PFC (BDNF mRNA: all F’s < 1.80, all *P*’s > 0.05, Fig. 4a. BDNF protein: all F’s < 1.35, all *P*’s > 0.05, Fig. 4b). Hypothalamic BDNF mRNA showed changes in sides (F_(1, 10)_ = 6.288, *P* = 0.0310) and interaction x sides (F_(1, 10)_ = 6.288, *P* = 0.0310) but no significant changes in condition (F_(1, 10)_ = 0.0110, *P* = 0.9184) was observed, Fig. 4c. BDNF protein levels in hypothalamus showed condition effect (F_(1, 8)_ = 5.55, *P* = 0.046) but no interaction effect between NS and S groups (F < 1.2, *P* > 0.05, Fig. 4d). CIS effect on TrkB and p-TrkB protein levels were unaltered in the PFC (TrkB: all F’s < 0.5, all *P*’s > 0.05, Fig. 5a and p-TrkB: all F’s < 2.7, all *P*’s > 0.05, Fig. 5b) and hypothalamus (TrkB: all F’s < 0.5, all *P*’s > 0.05, Fig. 5c and p-TrkB: all F’s < 1.1, all *P*’s > 0.05, Fig. 5d). These results demonstrate that CIS might not have affected BDNF system of PFC and hypothalamus in uninjured rats.

### Combined effects of CIS and thermal injury on BDNF mRNA and protein expression in the PFC and hypothalamus

**Fig. 6:**
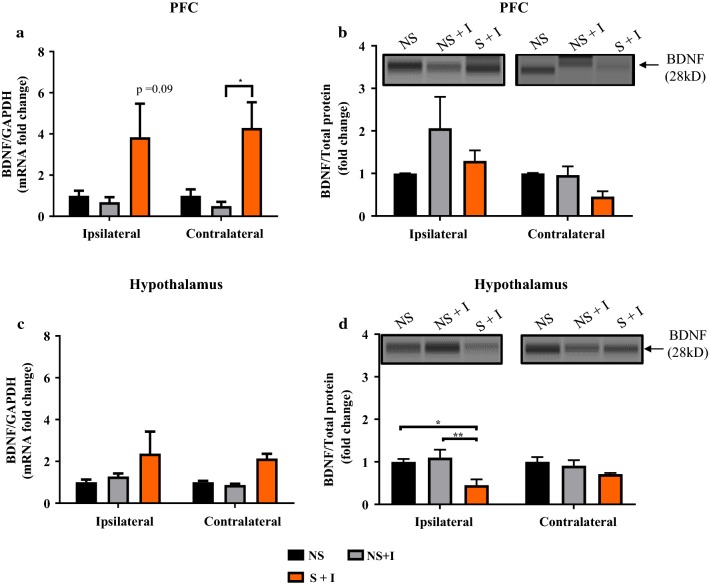
Effects of chronic stress and thermal injury on PFC and hypothalamic BDNF mRNA and protein level. Although the combined effects of chronic stress and thermal injury elevated ipsilateral and contralateral PFC BDNF mRNA expression, significant differences between groups (NS + I vs. S + I) was observed in the contralateral PFC (**a**). Ipsilateral and contralateral hypothalamus BDNF mRNA expression was not significantly different between experimental groups (**c**). BDNF protein level in the ipsilateral and contralateral PFC and hypothalamus are shown by Simple Western blot representative image (above quantification graphs of **b**, **d**). BDNF protein level was comparable among experimental groups in ipsilateral and contralateral PFC (**b**). BDNF protein was significantly decreased in the ipsilateral hypothalamus of S + I group in comparison to NS and NS + I groups (**d**). *NS* non-stress, *S* stress; *I* injury. n = 5/group. * = *P* < 0.05, **P < 0.01

After the final behavioral assessment (Fig. 1), the PFC and hypothalamus from both ipsilateral and contralateral sides to the injury were analyzed for changes in BDNF mRNA and protein expression using the RT-PCR and Simple Wes methods. Two way ANOVA analysis of BDNF mRNA of PFC showed significant difference in condition (F_(2, 22)_ = 8.769, *P* = 0.0016) but there was no noticeable changes between sides and interaction of condition × side (All F’s < 0.07 and all *P*’s > 0.05), Fig. 6a. Within the contralateral sides of PFC, S + I group showed more than three-fold increase in BDNF mRNA expression compared to NS + I and NS groups. However, a significant difference was observed between NS + I and S + I groups (Fig. 6a, *Post hoc* test *P* < 0.05). On the ipsilateral PFC even though S + I showed a higher level of BDNF mRNA expression in comparison to NS and NS + I groups it was not of significance (Fig. 6a, *P* = 0.09). A significant difference in BDNF protein levels between ipsilateral and contralateral sides was observed (F_(1, 12)_ = 5.63, *P* < 0.05) but there was no interaction (F < 1.51, *P* < 0.05) and condition (F < 2.1, *P* < 0.05) effect, Fig. 6b).

Ipsilateral and contralateral hypothalamus showed increased BDNF mRNA expression in S + I group in comparison to NS and NS + I groups but there was no significant difference among groups (Sides and interaction effects F’s < 0.3, *P* > 0.05; condition (F_(2, 22)_ = 4.346, *P* < 0.05, Fig. 6c). Ipsilateral hypothalamic BDNF protein level was significantly lower in S + I group compared to NS + I and NS groups (*P* < 0.05), but no significant changes in contralateral BDNF level among experimental groups was observed (condition effect: F_(2, 12)_ = 8.022, *P* = 0.006; side effect: F_(2, 12)_ = 0.058, *P* > 0.05; interaction effect: F_(2, 12)_ = 1.761, *P *> 0.05 = 0.2136, Fig. 6d). These data inform that BDNF mRNA expression is altered in the PFC but not in the hypothalamus whereas BDNF protein level in the hypothalamus are altered but not in the PFC by the combined effects of stress and injury.

### Combined effects of CIS and thermal injury on TrkB and p-TrkB levels in the PFC and hypothalamus

**Fig. 7:**
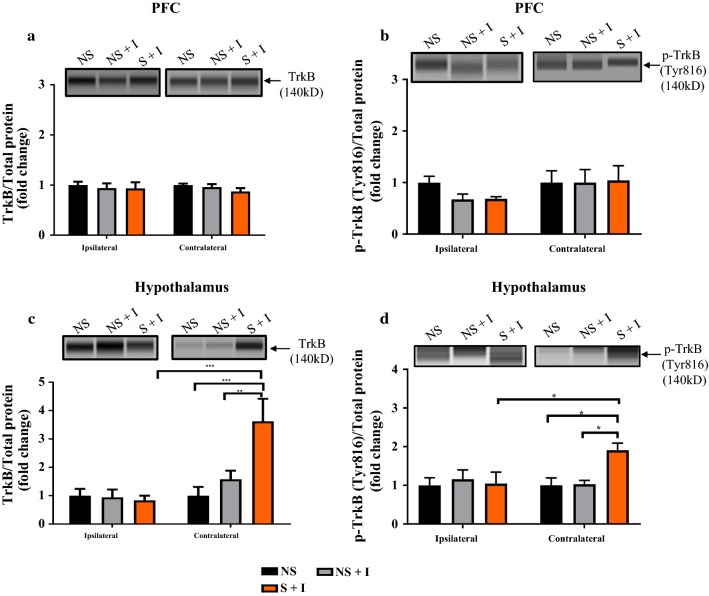
Effects of chronic stress and thermal injury on PFC and hypothalamic TrkB and p-TrkB levels. p-TrkB and TrkB protein levels in the ipsilateral and contralateral PFC and hypothalamus are shown by Simple Western blot representative image (above quantification graphs of **a**–**d).** p-TrkB and TrkB protein level was comparable among experimental groups in ipsilateral and contralateral PFC (**a**, **b**). p-TrkB and TrkB protein was significantly increased in contralateral hypothalamus of S + I group in comparison to NS and NS + I groups and also compared to ipsilateral S + I group (**c**, **d**). *NS* non-stress, *S* stress, *I* injury. n = 5/group. * = *P* < 0.05 and *** = *P* < 0.001

To determine whether CIS-induced increased mechanical allodynia is associated with changes in total TrkB and p-TrkB protein levels in the PFC and hypothalamus, we used Simple Western Protein analysis method. In the PFC, ipsilateral and contralateral TrkB and p-TrkB levels were unaffected in thermally injured rats with or without prior exposure to CIS as evidenced by no significant difference among NS, NS + I and S + I groups (Two-way ANOVA: TrkB: all F’s < 0.7, all *P*’s > 0.05, Fig. 7a; pTrkB: all F’s < 1.5, all *P*’s > 0.05, Fig. 7b). Contralateral hypothalamic TrkB and p-TrkB levels were significantly high in S + I group compared to NS + I and NS groups (Two-way ANOVA: TrkB: F_(2, 21)_ = 5.694, *P* = 0.0106; F_(1, 21)_ = 13.62, *P* = 0.0014; F_(2, 21)_ = 7.272, *P* = 0.0040; Fig. 7c; p-TrkB: F_(2, 18)_ = 2.63, *P* = 0.096; F_(1, 18)_ = 1.877, *P* = 0.187; F_(2, 18)_ = 3.298, *P* = 0.06 for the condition, side, and for the interaction condition × side, respectively, Fig. 7d). *Post hoc* test on TrkB revealed differences between S + I and NS + I (*P* < 0.01), and S + I and NS (*P* < 0.001), Fig. 7c) whereas for p-TrkB the difference between S + I and NS + I (*P* < 0.05), and S + I and NS (*P* < 0.05), Fig. 7d. Additionally, compared to ipsilateral, contralateral hypothalamic TrkB and p-TrkB levels were significantly higher in S + I group (TrkB, Fig. 7c, *P* < 0.001 and p-TrkB, 7D, *P* < 0.5). In ipsilateral hypothalamus TrkB and p-TrkB levels were comparable among experimental groups (Fig. 7c, d, *P* > 0.05). These results suggest that hypothalamic TrkB and p-TrkB levels were influenced by combined effects of CIS and thermal injury but not due to individual effects induced by CIS or thermal injury.

### TrkB receptor antagonist, CTX-B, treatments attenuate chronic stress-induced enhanced mechanical allodynia in thermally injured rats

**Fig. 8:**
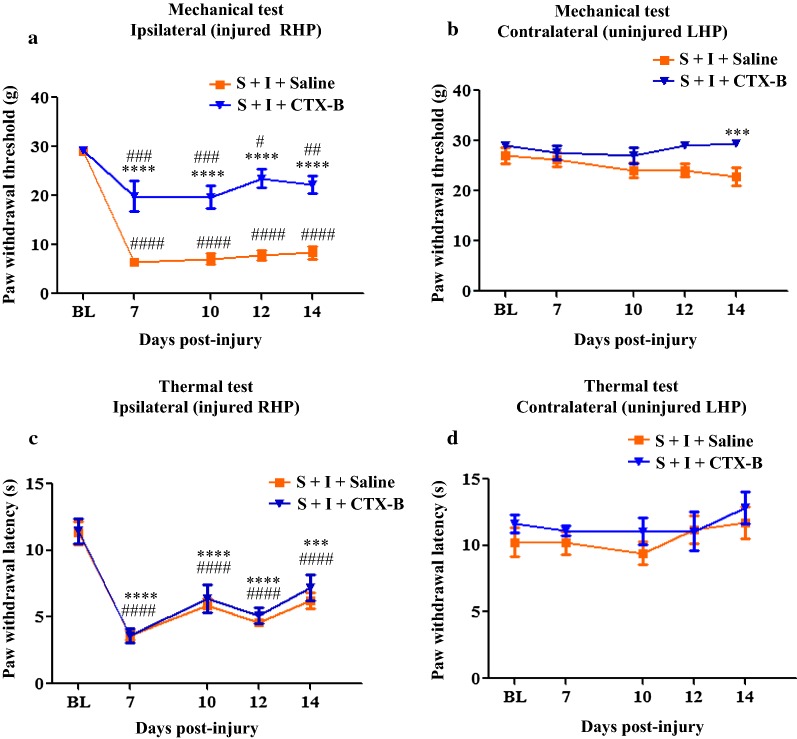
Effect of CTX-B on mechanical and thermal allodynia in stressed rats with thermal injury. CTX-B administration for 1–8 days significantly attenuated stress-induced enhanced mechanical allodynia in thermal injured rats at all times of testing. Both S + I + Saline and S + I +CTX-B groups showed significant reduction in PWT when compared to their respective baseline threshold on behavioral testing days 7–14. However, the S + I + Sal treated group showed lower PWTs on post-injury days 7–14 compared to the S + I + CTX-B group. **** = *P* < 0.0001 indicates significant differences between S + I + Sal and S + I + CTX-B treated groups. ^#^ = *P* < 0.05, ^##^ = *P* < 0.01, ^###^ = *P* < 0.001 ^####^ = *P* < 0.0001 indicates significant difference in comparison to their respective baseline threshold (**a**). In the contralateral paw, CTX-B-treated group showed increased withdrawal threshold compared to saline-treated group on post-injury day 14. *** = *P* < 0.001 indicates significant difference between S + I + Sal and S + I + CTX-B treated groups (**b**). No significant change in PWL was observed between S + I + Sal and S + I + CTX-B-treated groups throughout the testing period. However, both groups showed significant reduction in withdrawal latency when compared to respective baseline paw withdrawal latency. ^####^ = *P* < 0.0001 indicates significant difference between S + I + Sal and their baseline latency; **** = *P* < 0.0001 and *** = *P* < 0.001 compared between S + I + CTX-B and baseline PWL (**c**). Contralateral PWL was comparable between S + I + Sal and S + I + CTX-B-treated groups (**d**). *NS* non-stress, *S* stress, *Sal* saline, *I* injury; CTX-B: cyclotraxin; n = 6/group. Data is represented as mean ± SEM

We examined involvement of BDNF/TrkB signaling in CIS-induced exacerbated mechanical allodynia by administering TrkB specific antagonist CTX-B (20 mg/kg, intraperitoneal, once/day for 8 days). Both S + I + Sal and S + I + CTX-B-treated rats showed mechanical allodynia in the ipsilateral thermally injured hind paw (decreased PWT compared to respective baseline threshold). However, the mechanical allodynia was significantly exacerbated in S + I + Sal-treated rats as compared with the in S + I + CTX-B-treated rats (two-way RM ANOVA: F_(1, 12)_ = 103.9, *P* < 0.0001; F_(4, 48)_ = 42.5, *P* < 0.0001; F_(4, 48)_ = 9.024, *P* < 0.0001 for the condition, time, and for the interaction condition × time, respectively Fig. 8a). Specifically, the mechanical allodynia was shorter in S + I + CTX-B-treated rats as evidenced that no significant mechanical allodynia observed on post-injury days 12–14 (*P* > 0.05, compared to its baseline threshold) whereas S + I + Sal-treated rats displayed persistent mechanical allodynia during post-injury days 7–14 (*P* < 0.001, compared to baseline values); (2) the PWT was significantly higher in S + I + CTX-B-treated rats than S + I + Sal rats on each day of behavioral testing (Fig. 8a, *P* < 0.001). CTX-B treatments also reduced the mechanical allodynia developed on the contralateral paws in stressed rats with thermal injury on post-injury days (F_(1, 34)_ = 11.4, *P* = 0.0019; F_(7, 238)_ = 3.333, *P* = 0.0021; F_(7, 238)_ = 2.59, *P* = 0.0019 for the condition, time, and for the interaction condition x time, respectively. *Post hoc* test showed significant differences between S + I + Sal and S + I + CTX-B on day 14 (*P* < 0.001), Fig. 8b. These results indicate that CTX-B can attenuate chronic stress-induced exacerbated mechanical allodynia in thermally injured rats.

There were no differences in the ipsilateral and contralateral baseline PWLs between S + I + Sal and S + I + CTX-B groups (Fig. 8c, d, *P* > 0.05). Thermal injury in the ipsilateral paw produced a significant thermal allodynia in both the groups (*P* < 0.01 and *P* < 0.001, compared to respective baseline values). CTX-B-treated rats showed comparable thermal nociceptive threshold to that of saline-treated rats during post-treatment and post-injury days (two-way RM ANOVA: F_(1, 34)_ = 11.4, *P* = 0.0019; F_(7, 238)_ = 3.333, *P* = 0.0021; F_(7, 238)_ = 2.59, *P* = 0.0019 for the condition, time, and for the interaction condition x time, respectively Fig. 8c). Contralateral PWL did not differ between S + I + Sal and S + I + CTX-B groups at any time of assessment (Fig. 8d). It appears that the nature of CIS procedure has no effect on thermal injury-induced thermal allodynia in rats.

We also determined CTX-B effects in non-stressed thermally injured rats. Compared to respective baseline values, a significant reduction in PWT (*P* <0.05, Additional file 1: Figure S1A) and PWL (*P < *0.05, Additional file 1: Figure S1C) were observed in rats from both groups NS + I + Sal and NS + I + CTX-B. However, CTX-B treatments (20 mg/kg, intraperitoneal, once/day for 8 days) did not attenuate mechanical or thermal allodynia induced by thermal injury (*P* > 0.05). CTX-B treatments also did not alter contralateral PWT (Additional file 1: Figure S1B) and PWL (Additional file 1: Figure S1D). This suggests that CTX-B is effective only in reducing CIS-induced exacerbated mechanical allodynia in thermally injured rats.

### Effect of CTX-B on TrkB and p-TrkB in the PFC and hypothalamus

**Fig. 9:**
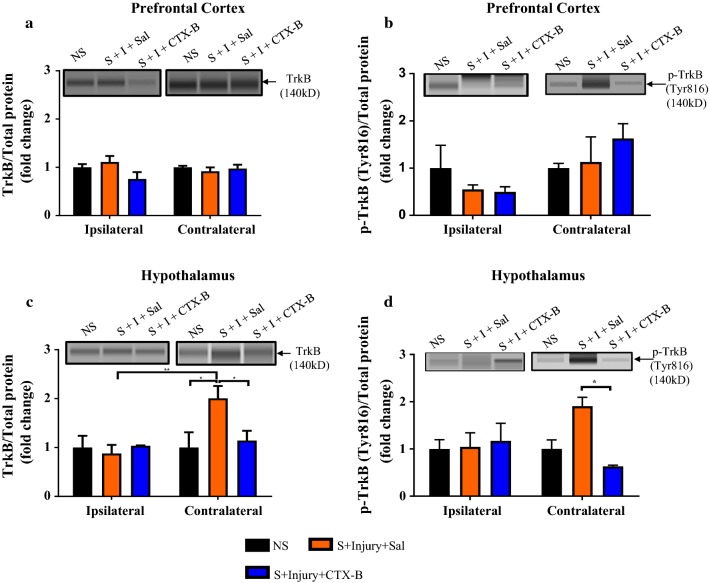
Effects of CTX-B on PFC and hypothalamic TrkB and p-TrkB levels in stressed rats with thermal injury. p-TrkB and TrkB protein levels in the ipsilateral and contralateral PFC and hypothalamus of NS, S + I + Saline and S + I + CTX-B treated rats are shown by Simple Western blot representative image (above quantification graphs of **a**–**d**). TrkB and p-TrkB levels in the ipsilateral and contralateral PFC were comparable between all groups (**a**, **b**). CTX-B treatment in the S + I group significantly reduced hypothalamic TrkB and p-TrkB levels compared to the S + I + Saline group (**c**, **d**). *NS* non-stress, *S* stress, *I* injury; *CTX-B* cyclotraxin-B, *Sal* saline. n = 5/group. * = *P* < 0.05 and ** = *P* < 0.01

We used simple Western analysis to examine the effect of repeated administration of CTX-B on TrkB and p-TrkB in the PFC and hypothalamus. Two-way ANOVA of TrkB and p-TrkB data revealed the following statistical results: PFC: No significant changes in expression of TrkB and p-TrkB among experimental groups (TrkB: all F’s < 2.3, all P’s > 0.05, Fig. 9a; p-TrkB: all F’s < 4, all P’s > 0.05, Fig. 9b). In hypothalamus, CTX-B treatments significantly reduced contralateral side TrkB and p-TrkB levels (TrkB: F_(2, 19)_ = 2.37, *P* = 0.121; F_(1, 19)_ = 5.33, *P* = 0.032; F_(2, 19)_ = 4.24, *P* = 0.03, Fig. 9c; p-TrkB: F_(2, 15)_ = 2.922, *P* = 0.0848; F_(1, 15)_ = 0.245, *P* = 0.627; F_(2, 15)_ = 3.89, *P* = 0.04, Fig. 9d for the condition, side, and for the interaction condition × side, respectively). No significant changes were observed among experimental groups on the ipsilateral side of the hypothalamus (Fig. 9c, d). These data suggest that CTX-B treatment has a more pronounced effect on hypothalamic TrkB and p-TrkB.

### Body weight

Rats from all experimental groups gained steady body weight during exposure to CIS procedure and also during post-injury days without noticeable significant differences among groups on the day of measurement (Additional file 1: Figure S2, *P* > 0.05). This suggests that neither CIS nor thermal injury influenced animal’s gain in body weight.

### Effect of CIS and thermal injury on c-Fos protein level in the PFC and hypothalamus

Simple Western analysis was used to measure c-Fos protein level as an indicator of neuronal activity in tissue samples. Two-way ANOVA showed no significant changes in PFC and hypothalamus c-Fos expression between ipsilateral and contralateral sides of NS and S groups (PFC: all F’s < 0.16, all *P*’s > 0.05, Additional file 1: Figure S3A; hypothalamus: all F’s < 2.7, all *P*’s > 0.05, Additional file 1: Figure S3B). Rats with injury alone and stress + injury showed no significant changes in c-Fos expression in PFC (PFC: Two-way ANOVA: all F’s < 3.85, all *P*’s > 0.05, Additional file 1: Figure S3C). In hypothalamus there was a condition effect but no difference in side and interaction effect (Two-way ANOVA: F_(2, 12)_ = 4, *P* < 0.05; F_(1, 12)_ = 2.926, *P* = 0.112; F_(1, 12)_ = 1.03, *P* = 0.388, Additional file 1: Figure S3D). These data suggest that there may be no ongoing neuronal activation in PFC and hypothalamus of post-stressed rats with and without thermal injury.

## Discussion

The results from this study demonstrate that CIS can induce transient mechanical allodynia in uninjured rats. Additionally, rats exposed to CIS exhibit exacerbated mechanical allodynia in the thermally injured state, and intraperitoneal administration of TrkB antagonist (CTX-B) attenuates it. In addition to these behavioral findings, stressed rats with thermal injury showed an augmented level of TrkB and p-TrkB in the hypothalamus and an increased BDNF mRNA expression in the PFC. CTX-B treatments reversed these changes. Collectively, these data indicate that there exists an association between chronic stress and mechanical allodynia in thermally injured rats and that the BDNF system in PFC and hypothalamus could play an important role in this relationship.

Here we examined the chronic cumulative effect of 4 different types of potential stressors on basal responses to mechanical and thermal stimuli. Our data (Fig. 2a) showed that CIS procedure does not modulate the PWL to thermal stimulus and PWT to mechanical stimulus in uninjured rats in the first 2 weeks of stress session: however, the rats start to develop increased sensitivity to non-noxious mechanical stimulus but not to thermal stimulus at third and fourth week following exposure to stress. CIS-induced altered PWT is transient as it was not evident during the post-stress period (Fig. 2b). We did not examine and compare the effect of individual stressor used in CIS procedure on the basal nociceptive threshold. However, previous studies report that rodents develop mechanical allodynia when exposed to a single type of stressor repeatedly. For example, Bardin et al. showed that exposure of uninjured rats to 1-h restraint stressor for 4 days/week over 5 weeks induces mechanical allodynia starting from the end of the first week of stress to the end of the period of stress, week 5, but not hyperalgesia. In contrast, exposure to forced swim stress for 8 days has no effect on hind paw mechanical sensitivity [22]. Similarly, short-term sleep disturbance-induced stress for 6 h daily for 3 consecutive days did not change basal responses to mechanical, heat, and cold stimuli [26]. Based on these reports and from our findings, it is clear that exposure of rodents to one or multiple types of stressors may or may not alter basal pain thresholds and it depends on the nature, intensity, and duration of exposure to stressors. Furthermore, the observed passing increase in mechanical allodynia in stressed rats may be due to stressors-induced mild anxiety associated nociception. Although we did not measure anxiety in the stressed animals, existing literature demonstrate altered pain behaviors in anxious animals [27, 28].

Burn pain is a subjective experience and it is influenced by physical, psychological and environmental factors [29]. Stress is found to modulate post-injury pain behaviors [4, 22, 30, 31]. Full and partial-thickness thermal injury at the mid-plantar surface of the rat hind paw induces intense pain behaviors at the site of injury and adjacent areas that last for 14 days post-injury [32, 33]. It is technically not feasible to assess stimulus-mediated evoked pain behaviors at the site of burn injury due to open wound/skin loss. Our behavioral data, Fig. 3a, showed that thermally injured rats that were prior exposed to CIS procedure display heightened, persistent mechanical allodynia at the proximal site to injury than non-stressed thermally injured rats. Furthermore, the mechanical allodynia spreads to the contralateral uninjured paw of stressed rats at the later stage of injury (post-injury days 10–14, Fig. 3b). It appears that CIS only affects mechanical but not thermal threshold as observed by the lack of significant changes in sensitivity to thermal stimulus between stressed and non-stressed animals with burn injury. Although the present study is the first to report that CIS play a critical role in the exacerbation of burn pain, previous studies have documented that physical and psychological stress delays recovery and worsens post-surgical pain [26, 34], enhances inflammatory pain [35], and heightens neuropathic pain symptoms [36, 37]. Taken together with the results of the present study, it is clear that there is a link between prior stress exposure and intensity of pain in the post-injury state. In general, stressors affect various forms of pain and it will be interesting to see if different types of stressors share a common ability to exacerbate post-injury pain symptoms.

We did not measure corticosterone levels in stressed rats with and without thermal injury. However, there are several studies showing stress-induced increased corticosterone levels in the plasma and brain tissue samples of stressed rodents [38–40]. In addition to CIS procedure-induced stress, pain behavioral tests might have influenced stress and corticosterone levels in rats. Further investigation will rule out whether multiple presentations of mechanical and thermal stimuli during experimentation contribute to the CIS-induced stress level.

PFC and hypothalamus are highly sensitive to stressors and modulate several behavioral and physiological responses to such events. BDNF and its receptor TrkB are highly expressed in both PFC and hypothalamus and their activity is altered following exposure to both stress and pain stimuli [16, 17, 41–43]. The role of the BDNF system in the PFC and hypothalamus in the post-stress state and in stress associated burn pain condition was unclear. Our results revealed that CIS had no effects on BDNF protein, BDNF mRNA, TrkB and p-TrkB expression levels in either left or right sides of the PFC and hypothalamus in uninjured rats (Figs. 4, 5). However, a significant increase in BDNF mRNA on the contralateral side of PFC (Fig. 6a) and reduced BDNF protein level in the ipsilateral hypothalamus (Fig. 6d) were observed in thermally injured rats with prior exposure to chronic stress. Furthermore, a significant increase in the expression of TrkB and p-TrkB in the contralateral side but not on the ipsilateral side of the hypothalamus of S + I group were observed (Fig. 7c, d). These findings suggest that compared to CIS or thermal injury alone their combination is effective in altering hypothalamic BDNF-TrkB system. Literature shows the discrepancy in the expression of BDNF and its receptor TrkB in brain regions following exposure to stress. For example, several studies have shown a stress-mediated decrease in BDNF mRNA and protein expression in the hippocampus [44, 45]. However, reports also show no significant changes in BDNF protein expression in the hippocampus of stressed animals [46, 47]. Some studies that have examined BDNF and TrkB in the PFC and hypothalamus in the stress state demonstrate upregulation and downregulation of them [48–51]. Furthermore, in the pain condition, BDNF is up-regulated in bodily fluids [52], dorsal root ganglia [53], cortex [9] and spinal cord [54]. Inflammatory pain up-regulates TrkB mRNA and protein expression in the dorsal horn [55]. In this study, BDNF, TrkB, and p-TrkB in the PFC and hypothalamus of NS + I rats was not altered compared to NS rats. This suggests that peripheral thermal injury-induced pain stimulus alone might not affect BDNF system of the PFC and hypothalamus.

Our findings along with earlier reports discussed above clearly indicate that the activity of BDNF and its receptor varies in brain regions which depend on the experimental stress procedure used in the respective studies. Additionally, previous studies measured BDNF and TrkB expressions during stressful or painful state [6, 55–57], whereas we analyzed BDNF and TrkB expressions on day 14 post-stress or post-injury state. This may be another reason for the discrepancy in results from the present study compared to earlier reports [55, 56, 58]. Furthermore, c-Fos protein levels in the PFC and hypothalamus showed no significant changes among experimental groups in both PFC and hypothalamus on post-stress/post-injury day 14 (Additional file 1: Figure S3). This evidence indicates that the stimuli (CIS or thermal injury or their combination) used in the present study is ineffective to induce neuronal activation until post-stress/post-injury day 14. Of note, we examined BDNF system in PFC and hypothalamus but further studies are warranted to investigate the regulation of BDNF system in other regions of the brain that may be sensitive in post-stress and post-burn states. Nevertheless, the present results along with existing studies clearly indicate a strong relationship between the BDNF system and stress associated pain.

Based on the findings that the combined effects of CIS exposure and peripheral injury alter BDNF system of PFC and hypothalamus, we hypothesized that BDNF-TrkB signaling participates in exacerbated mechanical allodynia. We tested this theory by administering TrkB specific antagonist CTX-B to S + I rats and assessed mechanical allodynia at multiple times. CTX-B attenuates aggravated mechanical allodynia in S + I rats indicating the involvement of BDNF-TrkB signaling in S + I condition. CTX-B had no effect on thermal allodynia (Fig. 8). It is important that the effect of CTX-B in blocking mechanical allodynia must be interpreted with caution because CTX-B might have also acted in different brain regions and also at the spinal level to abrogate mechanical allodynia in stressed rats with thermal injury. The spinal BDNF system was not examined in the present study because the aim was to establish a link between prior CIS exposure and burn pain mechanisms, with the PFC and hypothalamus being the relevant neural substrates for modulating both stress and pain. Moreover, previous studies have already shown the involvement of spinal BDNF system in different pain states [9, 59].

One study has demonstrated that CTX-B inhibits cold allodynia in the experimental trigeminal neuropathic pain model [59]. Surprisingly, our data showed that CTX-B treatments failed to reverse thermal injury-induced mechanical and thermal allodynia in rats (Additional file 1: Figure S1). One possibility is that burn injury might have activated multiple pain signaling pathways such as cytokines and adenosine triphosphate (ATP) in addition to BDNF system. This might be one reason that CTX-B might be ineffective in attenuating burn injury-induced pain behaviors but it is effective in reducing CIS-induced exacerbated mechanical allodynia. Furthermore, our data showed a reduction in the contralateral TrkB and p-TrkB level in the hypothalamus of stressed rats with the thermal injury following CTX-B treatments (Fig. 9c, d) indicating that hypothalamic BDNF-TrkB signaling plays an important role in chronic stress and burn pain induced exacerbation of mechanical allodynia.

## Conclusion

Taken together, the results from this study indicate that there exists a relationship between prior exposure to chronic stress and heightened post-burn pain and that the BDNF system in PFC and hypothalamus could play a significant role in this relationship. Although additional studies are necessary to investigate in detail the signaling mechanisms underlying the effect of CTX-B, it can be concluded that blockade of TrkB receptor could respite stress associated with heightened mechanical allodynia in burn injury state.

## Methods

### Animals

This study used a total of 60 adult male Sprague-Dawley rats of seven-eight weeks old purchased from the Charles River Laboratories, USA. Rats were quarantined for 3 days upon their arrival at our facility and then pair housed in cages with a 12 h light/dark cycle (6 am–6 pm) with free access to food and water. The rats spent a week in the vivarium before they were subjected to experimental procedures. The body weight of rats was in the range of 255–275 g before exposure to procedures and reached 430–460 g on the day of euthanization. All procedures were approved by the US Army Institute of Surgical Research (USAISR) Institutional Animal Care and Use Committee (IACUC). This study was conducted in compliance with the Animal Welfare Act, by implementing Animal Welfare Regulations and the principles of the Guide for the Care and Use of Laboratory Animals. Measures were taken to minimize the number of animals to be used for this study.

### Experimental groups

All experiments were performed in a blinded fashion. Figure 1 illustrates the scheme of experiments. We used four groups of the rat to study the effect of chronic intermittent stress (CIS) on nociceptive behaviors in uninjured and thermally injured rats. Group 1 was no stress (NS, n = 9), group 2 was stressed (S, n = 9), group 3 was no stress with thermal injury (NS + I, n = 9), and group 4 was stressed with thermal injury (S + I, n = 9). Four additional groups were used to investigate the involvement of BDNF-TrkB signaling in stressed rats with thermal injury. Group 5 was stressed, thermally injured, and received saline treatments (S + I + Sal, n = 6), group 6 was stressed, thermally injured and administered CTX-B treatments (S + I + CTX-B, n = 6) and groups 7 and 8 were non-stressed, thermally injured and received either saline or CTX-B (NS + I +Sal and NS + I + CTX-B). Saline (0.5 ml) or CTX-B (20 mg/kg in 0.5 ml) was injected once/day intraperitoneally for 8 consecutive days beginning from day 7 to day 14 post-injury.

### CIS procedure

Rats from all experimental groups were first acclimatized to the behavioral testing room, handling, sound chambers, empty glass cylinders (forced swimming test apparatus) and to the Plexi glass chambers of von Frey and thermal test devices for 4 days. The habituation time to each of the apparatus was 15 min. Following acclimatization, rats from groups 2, 4, 5 and 6 were presented with one type of stressor per day for 4 days (Monday–Thursday): (1) sound stimulus for 30 min; (2) restraint stimulus for 4 h; (3) cold stimulus for 4 h; and (4) forced swim procedure for 15 min. This procedure was performed over four weeks and the order of the stressors was changed weekly (Table 1). Sensitivity to mechanical and thermal stimuli was assessed before (on Monday) and 24 h after exposure to stressors (on Friday). During the 4 weeks of CIS procedure, the rats were not tested for pain sensitivity or exposed to stressors on Saturday and Sunday (Fig. 1). Control rats (groups 1 and 3) were not exposed to stressors but they were tested for mechanical and thermal sensitivity along with the stressed rats. After the 4 weeks of stress sessions, rats from groups 4, 5 and 6 were inflicted with thermal injury.

**Table 1:** Timeline of exposure to specific stressor per week and day

Week	Day	Stressor
1	Monday	Restraint stress (RS)
1	Tuesday	Forced swim stress (FSS)
1	Wednesday	Sound stress (SS)
1	Thursday	Cold stress (CS)
2	Monday	FSS
2	Tuesday	SS
2	Wednesday	CS
2	Thursday	RS
3	Monday	CS
3	Tuesday	SS
3	Wednesday	RS
3	Thursday	FSS
4	Monday	SS
4	Tuesday	RS
4	Wednesday	FSS
4	Thursday	CS

### Sound stress (SS) protocol

Rats were subjected to sound stress as described previously [23]. Briefly, rats were placed in an acrylic enclosure (8″ × 3 1⁄2″) contained in an acrylonitrile butadiene styrene (ABS) isolation chamber (Startle Response System apparatus, SR-Labs; San Diego Instruments, model numbers SIC002650-SIC002655) and habituated to the chamber for 20 min followed by exposure to 105 dB tone with frequencies ranging from 11 to 19 kHz, each lasting for 5–10 s randomly each minute over a total of 30 min period. Rats from the no stress group (control) were placed in the same testing chamber for 50 min but without exposure to the sound stimulus. Animals were returned to their home cages after sound or sham sound stress procedure.

### Forced swim stress (FSS) procedure

As shown in Fig. 1, rats were first acclimatized to the empty glass cylinder swim chamber (60 cm height × 25 cm diameter) for 15 min. On experimental days, individual rats were subjected to forced swim in the glass cylinder containing tap water at 30 cm depth at 25 ± 2 °C for 15 min. After the stress session, rats were dried with towels and returned back to their home cages. The water was changed for each of the rats subjected to forced swim. The control rats were placed in the glass cylinder without water and allowed to explore for 15 min.

### Cold stress (CS) procedure

The cold stress was induced by exposing pair housed rats to 4 °C for a period of 4 h. During the cold exposure, they had free access to food and water. Control rats remained in the behavioral testing room for 4 h.

### Restraint stress (RS) protocol

Individual rats were restrained for 4 h (from 9 a.m. to 1 p.m.) by placing in a well-ventilated Plexiglass tube (internal diameter, 3 cm; length, 11.5 cm, Harvard Apparatus) without food or water. The length of the tube was adjusted with a piston so that the animal was unable to move. Control rats remained in their home cages in the behavioral room.

### Induction of thermal injury (TI)

To induce thermal injury, individual rats were deeply anesthetized (3–4% isofluorane in oxygen) and a pre-heated (100 °C) soldering tip was placed on the mid-plantar surface of the right hind paw for 30 s [23, 32, 60]. This procedure produces full-thickness thermal injury and within 48 h secondary mechanical and thermal allodynia develops and lasts for 14 days post-injury [32]. The post-injury care was performed as described previously [23]. Briefly, immediately following induction of thermal injury, to minimize infection, silver sulfoxinide was applied one time to the site of injury. Wound assessment and the animal’s general appearance were observed during the experimental period as recommended by our Institutional Animal Care and Use Committee.

### Nociceptive behaviors testing

As shown in Fig. 1, changes in mechanical and thermal sensitivities were performed using von Frey and thermal apparatus at multiple times. In uninjured rats, paw withdrawal responses to mechanical and thermal stimuli were measured at every week before exposure to a stressor and 1 day after exposure to stressors. Behavioral testing in uninjured and injured rats without treatments was continued at multiple times during post-stress/post-injury days 3–14 (Figs. 2, 3). The thermally injured rats that received saline or CTX-B injections were tested 1 h after treatment on day 7, 10, 12 and 14 post-injury (Fig. 8 and Additional file 1: Figure S1). Baseline measurements were performed prior to CIS protocol and induction of thermal injury.

### Thermal nociceptive test

Rat hind paws sensitivity to thermal stimulus was measured using a thermal hyperalgesia instrument (Model 390; IITC Life Science, Woodland Hills, CA, USA). Rats were first acclimatized to the behavioral testing room (30 min) and to the Plexiglas chambers (20 min) immediately prior to testing. The instrument’s radiant heat source was focused on the plantar surface of the uninjured hind paw or on the adjacent, proximal area to the injury site of the injured hind paw until the animal withdraws its paw. The time between the application of thermal stimulus and the response time to remove the paw from the noxious stimulus was recorded as the Paw Withdrawal Latency (PWL). The intensity of the beam was set to 40% to produce a baseline PWL of approximately 10–12 s in naïve rats. A cut-off of 20 s was applied to avoid tissue damage. Three trials for each hind paw, with an interval of 5 min, were averaged and used for the analysis. The PWL scores from both left and right uninjured paws were combined to yield the mean PWL (Fig. 2c, d). Ipsilateral (injured) and contralateral (uninjured) paws withdrawal latencies from respective experimental groups were compared.

### Mechanosensitivity assay

Mechanosensitivity in response to a non-noxious stimulus was assessed in uninjured and injured rats using the Dynamic Plantar Anesthesiometer (Ugo Basile; Collegeville, PA). Briefly, a rigid von Frey tip with increasing force was presented at the plantar surface of the hind paw or at the adjacent, proximal site of the thermal injury until the rat withdraws its hind paw. The force required to elicit a paw withdrawal response was recorded as Paw Withdrawal Threshold (PWT; g). A cut-off value of 30 g was used to prevent tissue injury. Each rat was tested on both the right and left hind paw 3 times and the average was taken. As stated under the thermal nociceptive test, the PWT from the left and right paws of the uninjured rats were combined to yield the mean PWT (Fig. 2a, b). For the thermally injured group, the PWT was recorded from the ipsilateral paw (injured) and contralateral paws; then the two readings were compared with their respective control group (Fig. 3a, b).

### Tissue isolation

After the final behavioral experiments (Fig. 1), rats were humanely euthanized by decapitation method in accordance with USAISR IACUC Policy: Use and Maintenance of Guillotine’s for Rodents. Briefly, rats without analgesics/anesthetics were restrained in a plastic Decapicone and were decapitated using a guillotine (Harvard Apparatus) by a trained person. This method allows to obtain intact brain tissue without chemical contamination. The brains were immediately removed, flash-frozen in liquid nitrogen and stored at − 80 °C until use. The whole intact frozen brain was placed in a pre-chilled rat brain slicing matrix (Zivic instruments) at 4 °C. The brain was maintained in a semi-frozen state and all dissections were completed prior to thawing. To obtain the hypothalamic region, a coronal section was taken from − 0.40 to − 4.30 mm from bregma. The left and right hemisphere was separated at the corpus callosum and 4 mm thick punches from the left and right hypothalamic region was taken [61]. For dissection of the frontal cortex we followed the dissection parameters as shown earlier [10]. Briefly, the frontal cortex was separated from the whole brain by cutting at the first appearance of the corpus callosum at bregma 0.70 mm. The ventral area containing the olfactory nuclei was removed; leaving the dorsal prefrontal cortex intact which was further separated into left and right hemispheric regions.

### Total RNA and protein isolation

To isolate RNA and protein, 4-(2-Hydroxyethyl) piperazine-1-ethanesulfonic acid (HEPES) based (20 mM HEPES; 1 mM EDTA; 40 units/mL RNAse inhibitor; mini complete protease inhibitor tablet) buffer was added to the dissected right (ipsilateral) and left (contralateral) dorsal prefrontal cortex and hypothalamic samples. Tissue was homogenized 2 × for 20 s, split into two separate tubes, and centrifuged at 14,000×*g* for 20 min at 4 °C. Tri-reagent was added to the supernatant of one set of tubes followed by RNA isolation using the Zymogen Directzol RNA miniprep kit (ZRC175939). RNA concentration was determined by Nanodrop instrument. The pellet for the protein isolate was solubilized in RIPA for 20 min on ice. Following another centrifugation step, the supernatant was subjected to bicinchoninic acid (BCA; Pierce) assay to determine protein concentration.

### Quantitative RT-PCR analysis

Reverse-transcription was performed using the iScript cDNA synthesis kit (Biorad Cat#: 1708890) following the manufacturer’s directions. PCR was performed using iQ Sybr green supermix (Biorad 170-8880). The following PCR primers were used: BDNF forward: 5′-AGTGATGACCATCCTTTTCCTTAC-3′ and BDNF reverse: 5′-CCTCAAATGTGTCAT-CCAAGGA-3′ [62]; Glyceraldehyde 3-phosphate dehydrogenase (GAPDH) forward: 5′-AATCCCATCACCATCTTCCA-′3 and GAPDH reverse: 5′-TGGACTCCACGACGTACTCA-′3. Relative ratios were calculated by the equation, ratio = (2^ΔCPtarget (control − sample^)/2^ΔCPreference (control − sample^)), adapted from, in which CP is the threshold cycle, the target is the transcript of interest, and the reference is GAPDH.

### Simple western protein analysis

Glycosylated TrkB (LSBio, cat#: LS-C48549, size: 140kD), BDNF antibody (ThermoFisher cat# 710306), c-Fos (Millipore cat# PC05), p-TrkB (Tyr816, LSBio cat# LS-C95153) and total protein expression was determined by Wes analysis protein simple SM-W004, DM-001, DM-TP01; following the manufacturer’s directions. Briefly, the 5X fluorescent master mix was prepared with 400 mM dithiothreitol (DTT) and 10X sample buffer. The biotinylated ladder was prepared with 10X sample buffer, 400 mM DTT, and deionized water, denatured for 5 min at 95 °C, and loaded into lane 1 of the pre-filled plate provided by protein simple. The prepared 5X fluorescent master mix was combined with lysate for a final protein concentration of 0.2 mg/ml. The TrkB primary antibody (1:50 dilution) and luminol-S/peroxide combined substrate was prepared and loaded onto the plate following the assay plate layout designed by protein simple. Data analysis was conducted utilizing the Wes and ImageJ software.

### Statistical analysis

We used GraphPad 7 (GraphPad Software, Inc., La Jolla, CA, USA) statistical software to analyze the data. Values are expressed as the mean ± standard error of the mean (SEM). To analyze changes in body weight, mechanical and thermal allodynia with or without drug treatment over a period of time two-way repeated-measure (RM) ANOVA was utilized within-subjects (i.e. repeated) factor, and the condition [stress (S) and no stress (NS) or stress + injury (S + I) and no stress + injury (NS + I) as the between-subjects factor]. RT-PCR and Western blot data were analyzed using two-way ANOVA (ipsilateral (right)/contralateral (left) and condition (NS and S or NS + I and S + I) as factors. Bonferroni *Post hoc* multiple comparison test was performed to clarify group differences, as needed. *P* values of < 0.05 were considered significant.

## Additional file

**Additional file 1: Figure S1.** **Effects of CTX-B on mechanical and thermal allodynia in non-stressed rats with thermal injury.** Saline and CTX-B treated groups showed a significant reduction of the ipsilateral PWT (**A**) and PWL (**B**) compared to their respective BL values but there was no significant difference between the groups at times of behavioral testing indicating that 8 days of CTX-B treatments has no effect on PWT and PWL. CTX-B treatment (8 days) had no significant effects on the contralateral PWT (**C**) and PWL (**D**) * = *P* < 0.05, ** = *P* < 0.01, **** = *P* < 0.0001 compared between NS + I + CTX-B and baseline. #### = *P* < 0.0001 compared between NS + I + Sal and baseline. NS: non-stress; CTX-B: cyclotraxin-B. n = 6/group. Data is represented as mean ± SEM. **Figure S2. Effects of CIS and thermal injury on body weight.** CIS exposure did not alter body weight gain before or after induction of thermal injury. NS: non-stress; S: stress; I: injury; n = 9/group. Data is represented as mean ± SEM. **Figure S3. Effects of CIS on uninjured and thermal injured rats PFC and hypothalamic c-Fos levels.** c-Fos protein levels in the PFC and hypothalamus are shown by Simple Western blot representative image (above quantification graphs of **A**–**D).** CIS exposure had no significant effect on right and left side c-Fos protein expression within and between NS and S groups in the PFC (**A**) and hypothalamus (**B**). c-Fos protein level was not significantly different between experimental groups in ipsilateral and contralateral PFC (**C**) or hypothalamus (**D**) 14 days post CIS exposure or thermal injury induction. NS: non-stress; S: stress; I: injury. n = 5/group.

## Abbreviations

BDNF: brain-derived neurotropic factor
BL: baseline
CIS: chronic intermittent stress
CTX-B: cyclotraxin-B
CS: cold stress
FSS: forced swim stress
GAPDH: glyceraldehyde 3-phosphate dehydrogenase
LHP: left hind paw
M: mechanical
NS: no stress
NS + I: no stress + injury
PFC: prefrontal cortex
p-TrkB: phosphorylated tropomyosin receptor kinase BPWT paw withdrawal threshold
PWL: paw withdrawal latency
RHP: right hind paw
RS: restrain stress
S: stress
SS: sound stress
S + I: stress + injury
SAL: saline
TI: thermal injury
TrkB: tropomyosin receptor kinase B
