# Supplementary material for: Involvement of brain-derived neurotrophic factor (BDNF) in chronic intermittent stress-induced enhanced mechanical allodynia in a rat model of burn pain
Source: BMC Neurosci. 2019 Apr 24;20:17. doi: 10.1186/s12868-019-0500-1 (PMC6480655; doi:10.1186/s12868-019-0500-1)
Supplement: Supplementary file 1 — Additional file 1: Figure S1. Effects of CTX-B on mechanical and thermal allodynia in non-stressed rats with thermal injury. Saline and CTX-B treated groups showed a significant reduction of the ipsilateral PWT (A) and PWL (B) compared to their respective BL values but there was no significant difference between the groups at times of behavioral testing indicating that 8 days of CTX-B treatments has no effect on PWT and PWL. CTX-B treatment (8 days) had no significant effects on the contralateral PWT (C) and PWL (D) * = P < 0.05, ** = P < 0.01, **** = P < 0.0001 compared between NS + I + CTX-B and baseline. #### = P < 0.0001 compared between NS + I + Sal and baseline. NS: non-stress; CTX-B: cyclotraxin-B. n = 6/group. Data is represented as mean ± SEM. Figure S2. Effects of CIS and thermal injury on body weight. CIS exposure did not alter body weight gain before or after induction of thermal injury. NS: non-stress; S: stress; I: injury; n = 9/group. Data is represented as mean ± SEM. Figure S3. Effects of CIS on uninjured and thermal injured rats PFC and hypothalamic c-Fos levels. c-Fos protein levels in the PFC and hypothalamus are shown by Simple Western blot representative image (above quantification graphs of A–D). CIS exposure had no significant effect on right and left side c-Fos protein expression within and between NS and S groups in the PFC (A) and hypothalamus (B). c-Fos protein level was not significantly different between experimental groups in ipsilateral and contralateral PFC (C) or hypothalamus (D) 14 days post CIS exposure or thermal injury induction. NS: non-stress; S: stress; I: injury. n = 5/group. [file 12868_2019_500_MOESM1_ESM.pptx]

## Slide 1
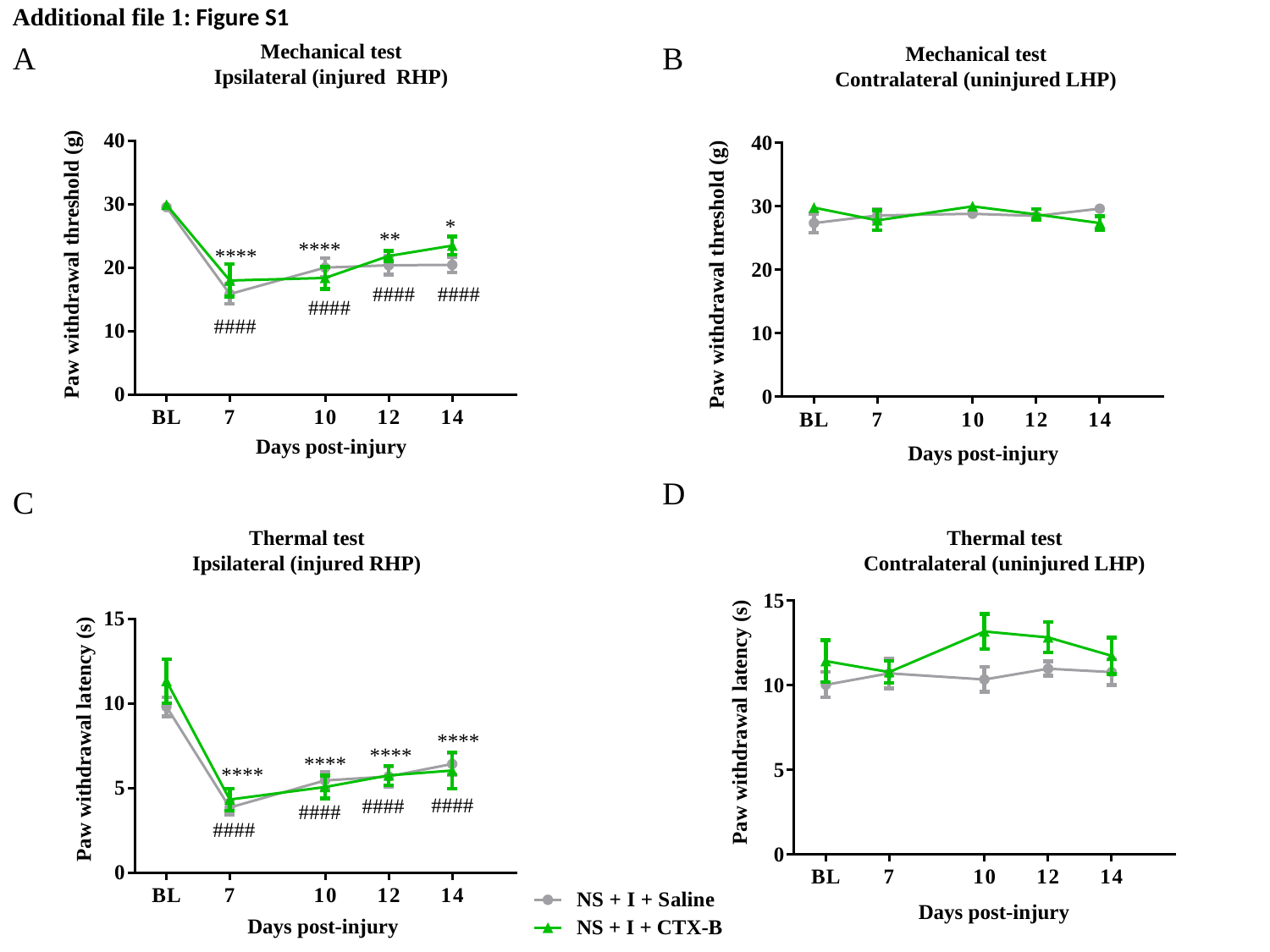

Additional file 1: Figure S1
A
Mechanical test
Ipsilateral (injured RHP)
B
Mechanical test
Contralateral (uninjured LHP)
Paw withdrawal threshold (g)
Days post-injury
*
**
****
****
Paw withdrawal threshold (g)
####
####
####
####
Days post-injury
D
C
Thermal test
Contralateral (uninjured LHP)
Paw withdrawal latency (s)
Days post-injury
Thermal test
Ipsilateral (injured RHP)
Paw withdrawal latency (s)
****
****
****
****
####
####
####
####
Days post-injury

## Slide 2
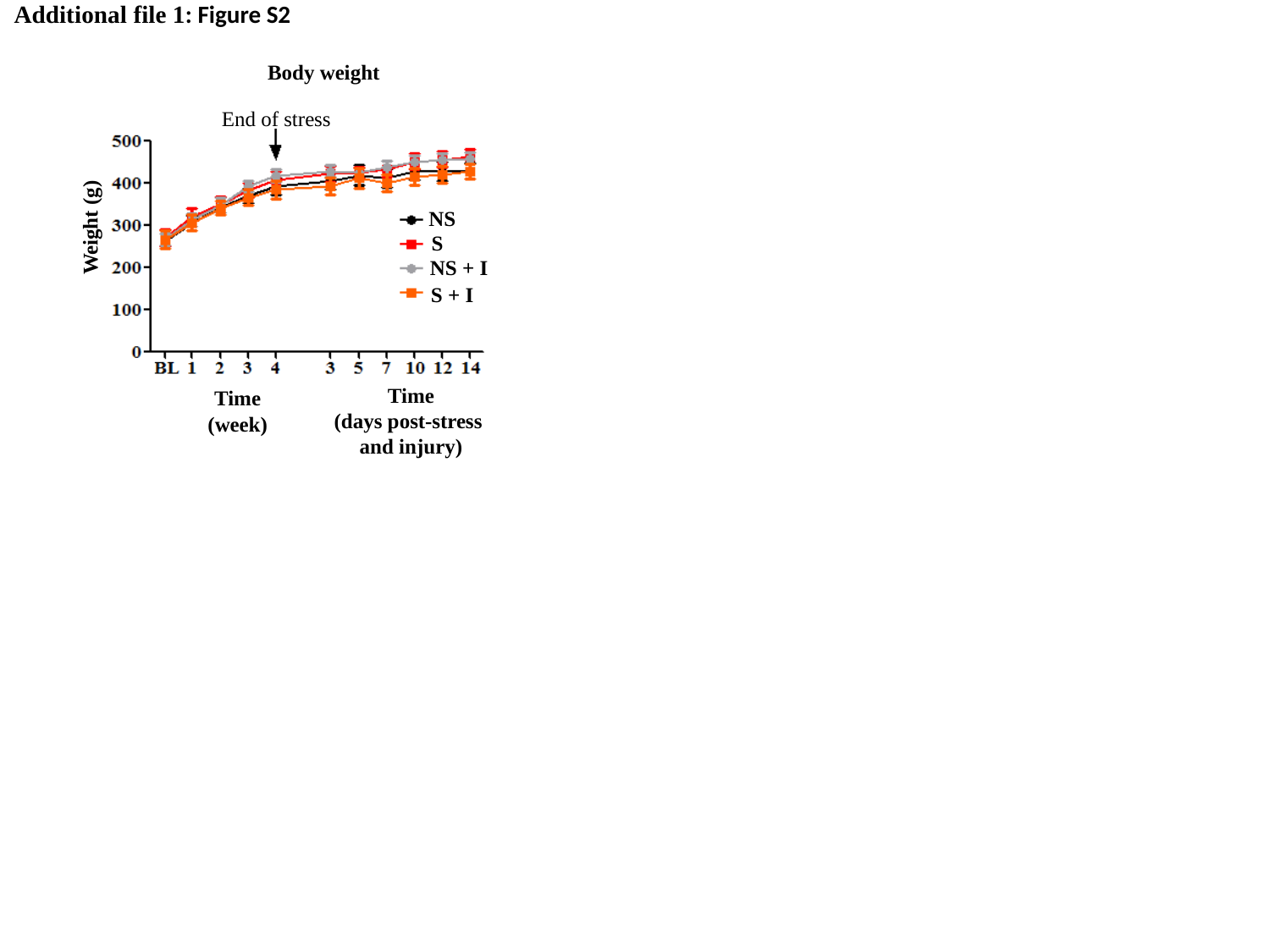

Additional file 1: Figure S2
Body weight
End of stress
Weight (g)
Time
(days post-stress
and injury)
Time
(week)
NS
S
NS + I
S + I

## Slide 3
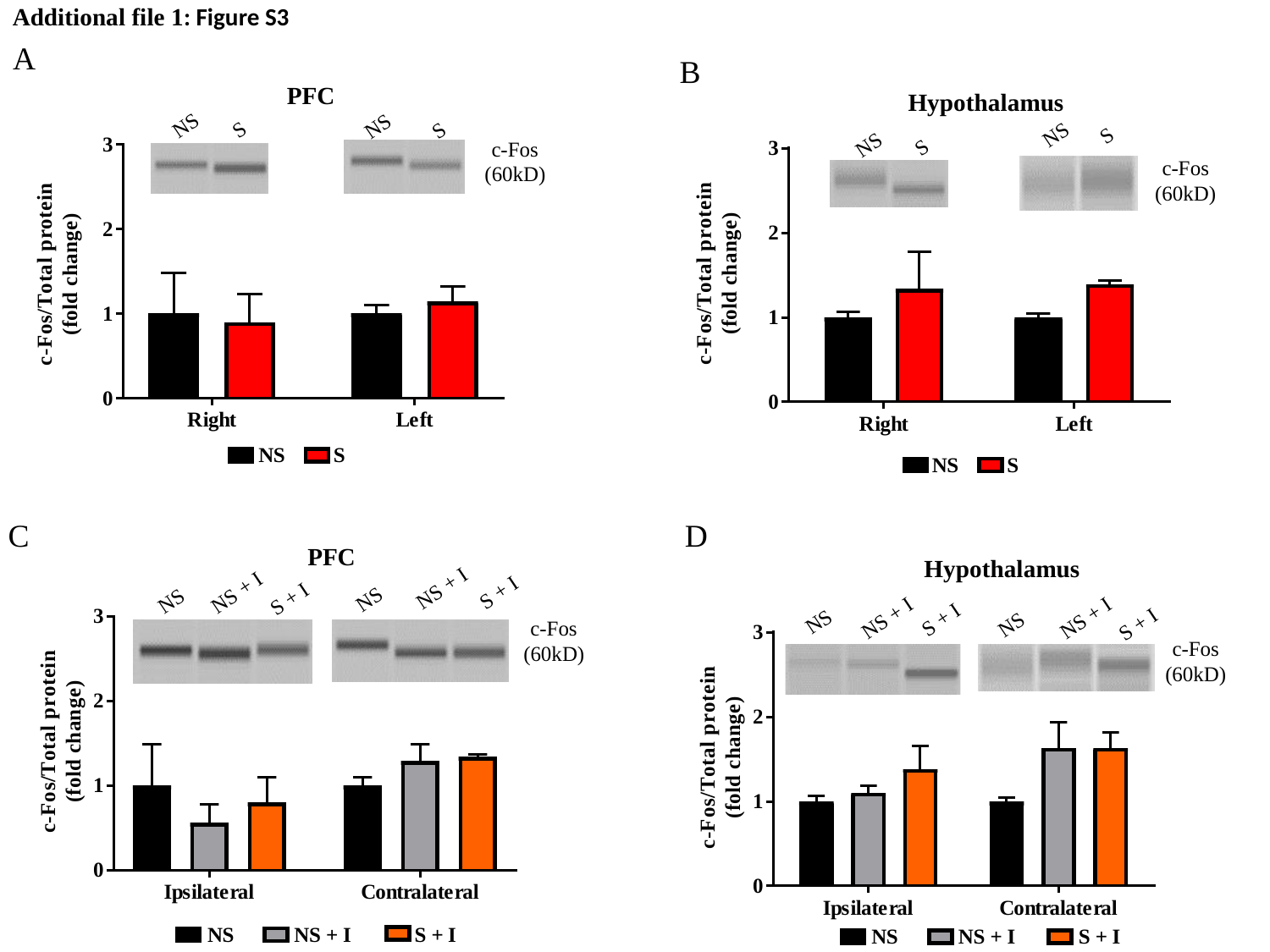

Additional file 1: Figure S3
A
B
PFC
Hypothalamus
NS
S
NS
S
c-Fos
(60kD)
NS
NS
S
S
c-Fos
(60kD)
C
D
PFC
NS + I
S + I
NS + I
S + I
c-Fos
(60kD)
NS
NS
Hypothalamus
NS + I
NS + I
S + I
S + I
c-Fos
(60kD)
NS
NS
